# Endotheliitis and Endothelial Dysfunction in Patients with COVID-19: Its Role in Thrombosis and Adverse Outcomes †

**DOI:** 10.3390/jcm9061862

**Published:** 2020-06-15

**Authors:** Wassim Mosleh, Kai Chen, Steven E. Pfau, Aseem Vashist

**Affiliations:** 1 Division of Cardiovascular Medicine, University of Connecticut, Farmington, CT 06105, USA; mosleh@uchc.edu (W.M.); kachen@uchc.edu (K.C.); 2 Division of Cardiovascular Medicine, VACT Healthcare System, West Haven, CT 06111, USA; steven.pfau@yale.edu; 3 Division of Cardiovascular Medicine, Yale School of Medicine, New Haven, CT 06510, USA; 4 Division of Cardiovascular Medicine, Saint Francis Hospital, Hartford, CT 06205, USA

Coronavirus disease 2019 (COVID-19) is a contagious disease caused by severe acute respiratory syndrome coronavirus-2 (SARS-CoV2), emerging in Wuhan, China and developing into a pandemic with rapidly emerging cardiovascular manifestations. In the United States, there are approximately 1.7 million reported cases with over 100,000 deaths as of 30 May 2020 [1]. Severe SARS-CoV-2 infection is more commonly observed in patients with specific comorbidities, including cardiovascular disease, diabetes and obesity, yet the mechanism of this relationship is unclear. SARS-CoV2 binds to the angiotensin-converting enzyme 2 (ACE2) receptor, which is abundantly expressed in human tissues including lung epithelium, myocardium, and vascular endothelium [2]. The virus has been shown to directly infect engineered human blood vessel organoids in vitro [3]. A rapidly accumulating body of evidence suggests that COVID-19 causes vascular derangements as a consequence of endothelial cell infection by the virus, contributing to observed cardiovascular and pulmonary complications.

Advanced age, hypertension, diabetes, smoking and coronary artery disease are risk factors for severe COVID-19, conditions which are all associated with vascular endothelial dysfunction. Recent reports have also shown a robust and independent association between obesity and the severity of COVID-19 infection, even in the absence of other co-morbidities [4,5]. Obesity is a chronic inflammatory state associated with dysregulated endocrine and paracrine actions of adipocyte-derived factor, which in turn disrupt vascular homeostasis and cause endothelial dysfunction. While the mechanisms through which obesity exacerbates COVID-19 infection are not fully understood, endothelial dysfunction may be the common link [6,7]. A prothrombotic state is evident in COVID-19 patients, with elevated D-dimer levels, arterial and deep venous thrombosis, pulmonary embolism, strokes, and intracardiac and microvascular thrombi. It is postulated that endothelial dysfunction and endotheliitis (inflammation of the blood vessel wall) result in thrombus formation. A new pulmonary complication called microvascular COVID-19 lung vessels obstructive thromboinflammatory syndrome (MicroCLOTS) has been described in severe COVID-19 and likely represents an atypical form of acute respiratory distress syndrome (ARDS) [8]. MicroCLOTS is a progressive, diffuse endothelial thromboinflammatory syndrome which is characterized by the development of microvascular pulmonary thrombosis. On a background of endothelial dysfunction, endotheliitis (endothelialitis) likely plays a central role in the development of COVID-19 related thromboembolic phenomenon and pulmonary injury [8].

Since the vascular endothelium is a dynamic endocrine, paracrine, and autocrine organ with a vital role in regulating vascular tone and homoeostasis [9], its dysfunction leads to detrimental shifts in the vascular equilibrium towards vasoconstriction (manifesting clinically as organ ischemia, infarction and intrapulmonary shunting), inflammation, and a pro-coagulant state resulting in thrombosis [10]. A recent study has shown that angiopoietin-2 is significantly elevated in critical COVID-19 patients, suggesting its role as a strong prognostic biomarker [11]. The notion of angiopoitein-2 being a marker of endothelial damage relates to its presence and release from endothelial Weibel–Palade bodies has reinforced the hypothesis of a COVID-19-associated microvascular dysfunction [11]. The presence of viral inclusion structures has also been demonstrated in endothelial cells, consistent with direct viral infection of these cells; histologic assessment of these specimens revealed endotheliitis of the submucosal vessels [12]. The authors theorize that the presence of viral elements in the endothelium, by inclusion via the ACE2 receptor, recruits immune cells, thereby resulting in widespread endothelial dysfunction associated with apoptosis. Similarly, an autopsy case series [13] demonstrated severe pulmonary endothelial injury associated with the presence of intracellular virus and disrupted cell membranes [13]. Histologic analysis of pulmonary vessels also showed widespread thrombosis with microangiopathy; alveolar capillary microthrombi were nine times as prevalent in patients with Covid-19 compared to patients with influenza. Similarly another study, which examined skin and lung tissues from COVID-19 patients, showed that the pattern of COVID-19 pneumonitis was predominantly a pauci-inflammatory septal capillary injury [14]. This was accompanied by significant deposits of terminal complement components C5b-9 (membrane attack complex), C4d, and mannose binding lectin (MBL)-associated serine protease (MASP)2 in the microvasculature [14]. This is consistent with the hypothesis that COVID-19 infection causes a calamitous microvascular injury syndrome which includes activation of complement pathways, endotheliitis and an associated thrombotic state [15].

Future studies will likely focus on the role of endothelial dysfunction and endotheliitis in the pathogenesis of COVID and its related complications. While there are several invasive (e.g., coronary epicardial vasoreactivity (QCA) using provocation testing with intracoronary acetylcholine) and non-invasive (e.g., flow-mediated-dilation and peripheral arterial tonometry) tools to assess endothelial dysfunction, serum biomarkers will likely emerge as surrogate evidence for endotheliitis [9].

Similarly, addressing endothelial dysfunction and inhibiting the inflammatory response will likely be the underpinnings of a successful preventive and therapeutic strategy. The observations of EC viral infection and subsequent injury provide a rationale for therapies aimed at stabilizing the endothelium with anti-inflammatory and immune-modulating drugs. Clinical use of inhibitors of complement such as eculizumab (anti-C5 monoclonocal antibody) and RUKONEST (C1 inhibitor) have been reported [16,17]. In addition to more widespread use of IL-6 inhibitors such as tocilizumab, TNF inhibitors [18], ACE inhibitors [19], statins [20] and endothelin receptor antagonists [21] are being actively pursued. In addition to the aforementioned inflammation-modulating therapy, another important approach to reversing endothelial dysfunction is to enhance the vasoprotective effect of nitric oxide (NO), which is well known to have a wide range of biological properties involving vasodilation, angiogenesis, and anti-thrombosis. In the setting of endothelial dysfunction, there is impaired nitric oxide bioavailability either by diminished production by endothelial nitric oxide synthase or excess oxidative degradation. Ongoing research is examining the use of inhaled NO in COVID-19 with dual effect as a pulmonary vasodilator and direct antiviral activity by interfering with S-protein-ACE-2 interaction [22,23]. NO is amongst the most vital vasodilator molecules, and also inhibits other important events in the development of platelet adhesion and aggregation [9]. Finally, the role of anticoagulation is being pursued aggressively given the development of venous and arterial thrombosis in these patients.

In conclusion, understanding the relationship between COVID-19, endotheliitis, pre-existing endothelial dysfunction, and observed endothelial injury will be imperative to developing new therapeutic targets to alter the trajectory of this pandemic. We also believe that the understanding of the relationship between endothelial health and susceptibility to severe COVID-19 and other infections may have implications for public health policy in the future.
